# Anaplastic Lymphoma Kinase (ALK)-Rearranged Renal Cell Carcinoma: A Case Report Highlighting Diagnostic Challenges and Therapeutic Opportunities

**DOI:** 10.7759/cureus.65621

**Published:** 2024-07-29

**Authors:** Eltayeb Elhassan, Corina Girleanu, Paul Kelly, Derek G Power, Paul Sweeney, Nick Mayer, Richard M Bambury

**Affiliations:** 1 Medical Oncology, University Hospital Kerry, Tralee, IRL; 2 Histopathology, Cork University Hospital, Cork, IRL; 3 Radiation Oncology, Bon Secours Hospital, Cork, IRL; 4 Medical Oncology, Cork University Hospital, Cork, IRL; 5 Urology, Mercy University Hospital, Cork, IRL

**Keywords:** renal cell carcinoma (rcc), nephrectomy, histopathology (hp), anaplastic lymphoma kinase, carboplatin, carcinoma, oncology, alectinib, kidney cancer

## Abstract

A 57-year-old male underwent an open right radical nephrectomy in 2015 for a 3-cm kidney tumor which was classified at the time as a combined tubulocystic and collecting duct carcinoma. One of six nodes was positive for metastatic carcinoma and the patient received adjuvant carboplatin/gemcitabine chemotherapy. In 2020, he developed enlarging retroperitoneal adenopathy and underwent a retroperitoneal lymph node dissection with 11 of 13 nodes in the resected specimen positive for the previously described renal carcinoma, followed by adjuvant radiotherapy. In November 2022, he again underwent surgery for further locoregional recurrence with resection of a right psoas mass lesion and right hemicolectomy. Pathology on this occasion was reclassified as anaplastic lymphoma kinase-rearranged renal cell carcinoma (ALK-RCC). Shortly afterward, a restaging CT revealed multiple liver metastases and evidence of further disease recurrence in the right renal bed. He commenced alectinib with a complete radiological response and has continued on it for 12 months at the time of writing this report.

To our knowledge, there are only five prior reports of ALK-RCC treated with targeted ALK inhibitor therapy in the literature. We report this case to highlight the importance of recognizing and diagnosing this rare RCC subtype since it has significant therapeutic implications. Furthermore, to our knowledge, this patient has had the longest follow-up reported to date in the literature so far. A concerted effort by the histopathology and oncology community is needed to gather more data on the incidence and treatment outcomes of these tumors so that progress can be made in optimizing their management. It is important to consider novel and emerging entities from the most recent WHO 2022 classification, many of which are defined by molecular characteristics with associated therapeutic implications.

## Introduction

The anaplastic lymphoma kinase (ALK) protein is a tyrosine kinase in normal cells, and ALK gene alterations can be potent drivers of oncogenes in a variety of tumor types including lung cancer and lymphoma. ALK tyrosine kinase inhibitors (ALK-TKI) have been successfully deployed as therapeutic modalities in the setting of these driver alterations in a variety of tumor types [1-4]. Renal cell carcinoma (RCC) has an annual incidence of approximately 10 cases per 100,000 in most Western countries [5]. The 2022 WHO pathological classification has confirmed clear cell RCC as the most common histopathological subtype [6]. However, there is a wide variety (>20 subtypes) of non-clear cell RCC, and ALK-rearranged RCC (ALK-RCC) is now included in the WHO classification system as one of these.

ALK-RCC is a novel and very rare subtype of molecularly defined RCC, accounting for an estimated 0.3% of RCCs as reported in an analysis of four large cohorts comprising 2195 cases of adult kidney cancer [7]. Another recent Turkish study aimed to detect ALK RCC in 276 patients with known non-molecularly defined RCC (both clear cell and non-clear cell cases were included). While ALK immunohistochemistry (IHC) was positive in eight patients, fluorescence in situ hybridization (FISH) analysis confirmed ALK rearrangement in only three of eight cases [8]. From a histopathological perspective, ALK-RCC is defined by heterogeneous and mixed morphology and has a wide differential diagnosis including fumarate hydratase (FH)-deficient RCC, SMARCB1-deficient renal medullary carcinoma, collecting duct carcinoma, papillary RCC, MiT family translocation RCC, and mucinous tubular and spindle-cell carcinoma (MTSCC) [9].

ALK-rearranged RCCs are known to have a variety of fusion partners including VCL, TPM3, EML4, STRN, and HOOK1. The VCL:ALK fusion is the most well-known, affecting young patients with sickle cell traits and having a distinctive morphology [10]. ALK-RCCs with fusion partners other than VCL show a more heterogeneous appearance [10]. IHC for ALK protein is a useful screening test for ALK-RCC; however, for an accurate diagnosis, ALK rearrangement should be confirmed by FISH or next-generation sequencing (NGS). In this report, we present the case of a patient who was retrospectively diagnosed with this novel entity through careful clinical and histopathologic correlation and demonstrate the associated therapeutic implications.

This article was previously presented at the Irish Society of Medical Oncology meeting on January 19, 2024.

## Case presentation

Clinical summary

In December 2014, a 57-year-old previously well male was investigated for frank hematuria. There was no clinical evidence to suggest sickle cell trait, and a thoraco-abdominal CT scan revealed a 3-cm lesion in the upper pole of the right kidney with a small associated aorto-caval lymph node. The findings were concerning for primary RCC with associated adenopathy but no evidence of distant metastases were seen. The patient underwent an open radical right nephrectomy and interaortocaval lymphadenectomy. Intraoperatively, six enlarged aortocaval lymph nodes were identified and surgically dissected. Histology showed a medullary tumor with a heterogenous morphology and prominent tubulocystic appearance, best classified at that time as a combined tubulocystic and collecting duct carcinoma - AJCC stage pT3aN1 (stage III). He was treated with four cycles of adjuvant carboplatin/gemcitabine postoperatively.

In 2019, a surveillance CT scan showed a solitary aortocaval node measuring 24 x 18 mm. A subsequent PET scan showed FDG-avid aortocaval and right retrocaval lymphadenopathy consistent with recurrent malignancy. The patient underwent retroperitoneal lymph node dissection. A recurrence of the patient’s prior collecting duct carcinoma of the kidney in 11 of 13 nodes was reported histologically. The procedure was technically difficult due to the proximity of the nodes to the large vessels of the abdomen and focally tumor cells extending very close (<0.1 mm) to the cauterized surface with tissue fragmentation. All gross disease was resected at surgery. He received adjuvant radiotherapy with 45 Gy in 25 fractions delivered to the para-aortic and interaortocaval region.

**Figure 1:**
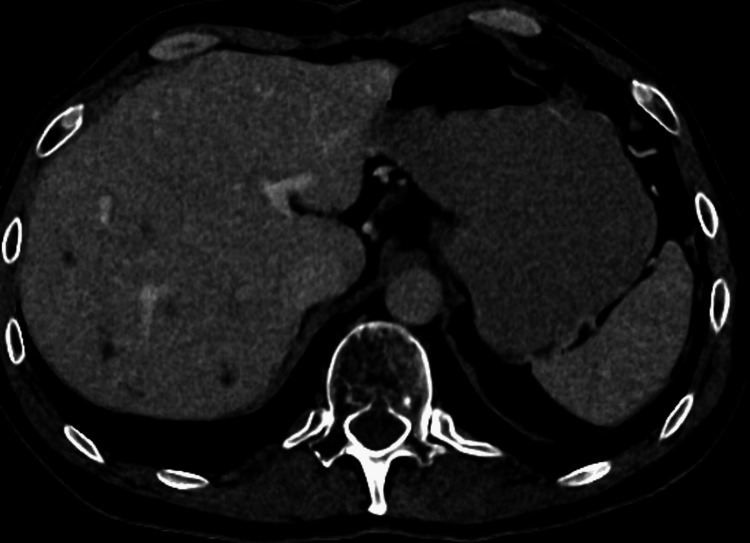
CT scan showing liver metastases prior to commencing alectinib CT: computed tomography

**Figure 2:**
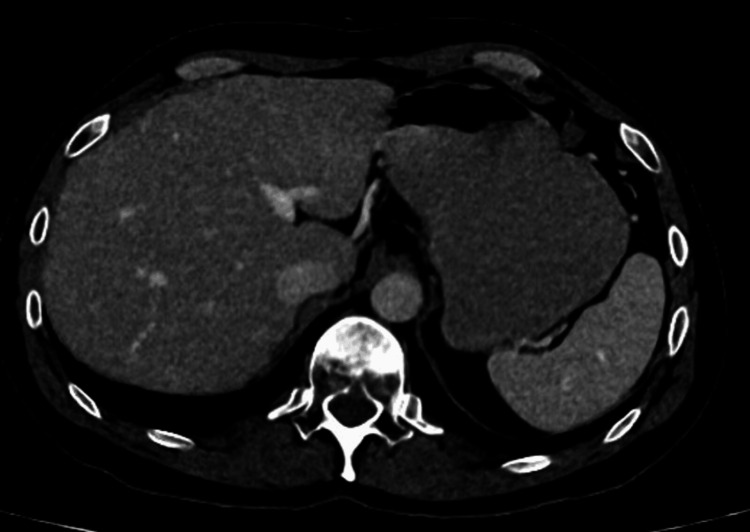
CT scan showing liver metastases after 12 months on alectinib CT: computed tomography

In March 2022, a surveillance CT scan showed a large recurrent necrotic mass in the right nephrectomy bed close to the psoas muscle, measuring 6 cm in keeping with further recurrence, without distant metastases. In November 2022, he underwent further surgery with resection of a right psoas mass lesion and synchronous right hemicolectomy due to the proximity of the mass to the right colon. Histopathology on this occasion showed similar morphology and immunoprofile to the previous right nephrectomy specimen. However, given the advances in the RCC classification and data in the recent literature, the case was referred externally for additional immunohistochemistry and molecular studies where ALK rearrangement was detected. The final diagnosis was ALK-RCC. Shortly afterward, a restaging CT revealed multiple liver metastases and evidence of further disease recurrence in the right renal bed (Figure 1). The patient was started on alectinib with complete radiological response, which had been ongoing for 12 months at the time of writing this report (Figure 2).

Pathological findings of resected specimens

**Figure 3:**
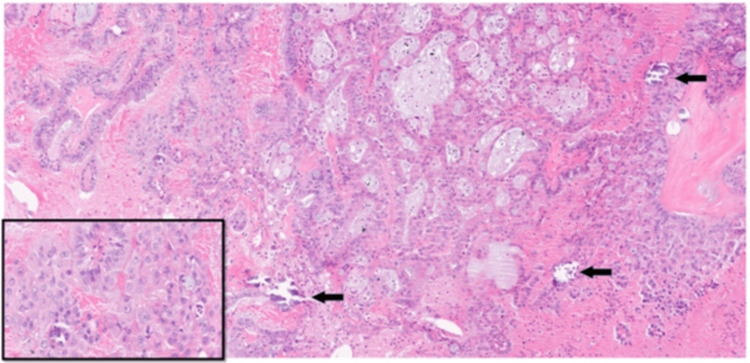
ALK-rearranged RCC showing papillary and cribriform architecture and psammomatous calcifications (black arrows) Inset: high-grade cytological features and prominent nucleoli ALK: anaplastic lymphoma kinase; RCC: renal cell carcinoma

**Figure 4:**
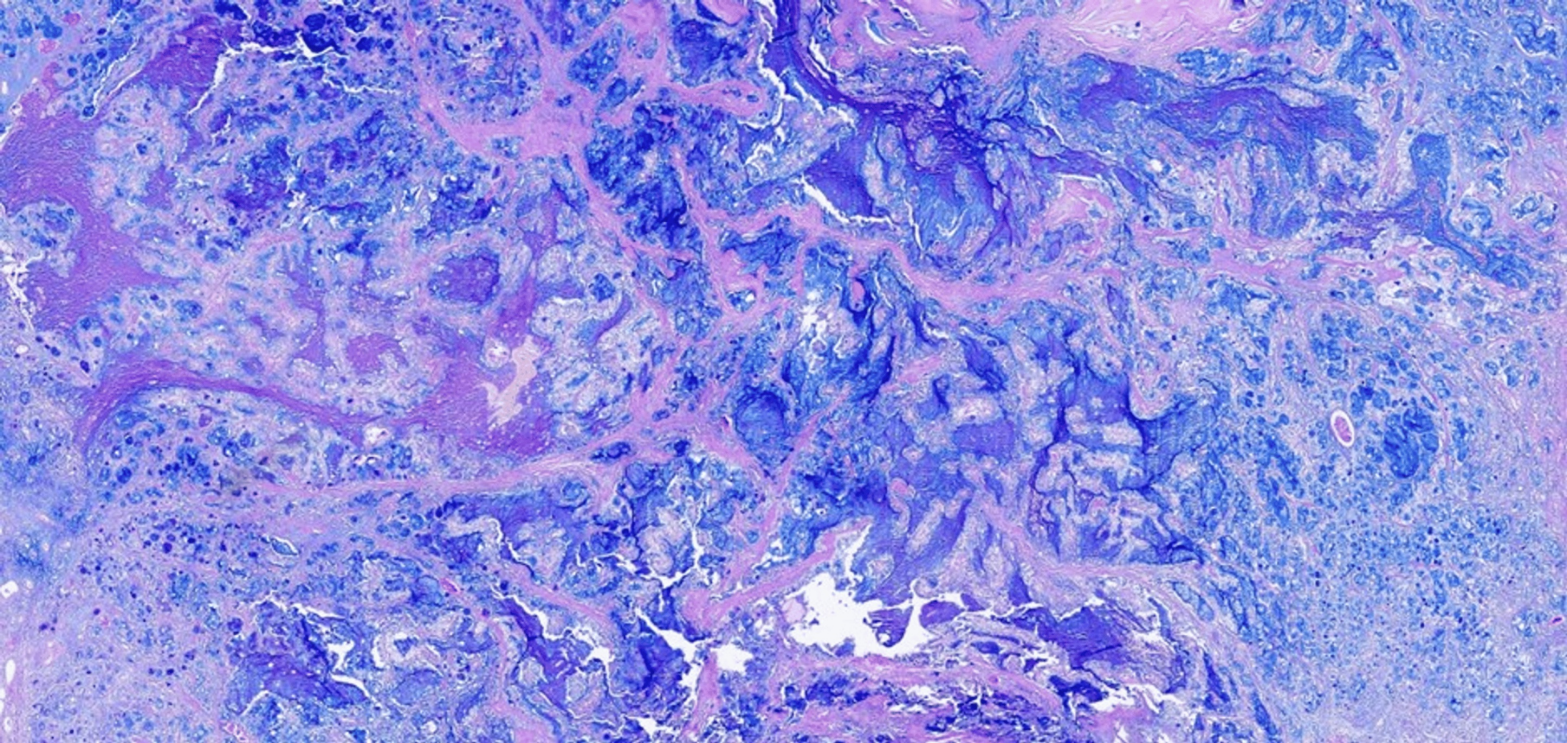
Alcian blue stain highlighting prominent extracellular mucin

The sections from the right nephrectomy specimen in 2015 showed a well-circumscribed, unencapsulated ISUP grade 3 tumor that was partly centered within the renal medulla and extending into the collecting system/renal pelvis and also showed perinephric fat invasion. Histologically, the tumor had a heterogenous appearance, with a tubulocystic, cribriform, and sieve-like architecture with a lesser papillary component (Figure 3). The papillae seen were branching with fibrovascular cores and were lined by a pseudostratified layer of columnar cells with focal high-grade cytological atypia and prominent nucleoli (Figure 3). There was also prominent mucin within the tumor and occasional psammomatous calcifications (Figure 4). Neither rhabdoid nor sarcomatoid changes were seen. The urothelium lining the collecting system was benign in appearance.

Immunohistochemically, the tumor was positive for PAX8, diffusely for CK7 and 34beta E12, with patchy positivity for p63 and CEA. INI-1 showed retained (normal) staining. The tumor was negative for AMACR and OCT 4. The presence of stromal mucin was confirmed on an ABPAS stain.

**Figure 5:**
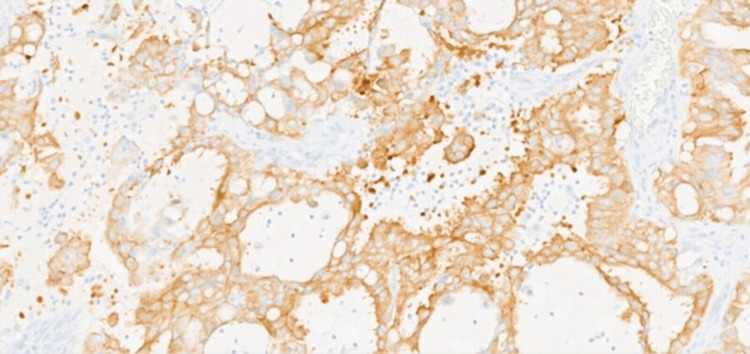
Diffuse membranous and cytoplasmic ALK-IHC staining ALK-IHC: anaplastic lymphoma kinase-immunohistochemistry

The diagnosis at that time was combined tubulocystic and collecting duct renal carcinoma. However, following the right psoas and right hemicolectomy resection in 2022, given the atypical clinical behavior for a collecting duct carcinoma, new entities described in the recent literature were considered. One of the main differential diagnoses was FH-deficient RCC, which can show a tubulocystic morphology. The case was therefore referred externally for further IHC and molecular investigations, which are not available in our laboratory. There was no evidence of deficiency of succinate dehydrogenase (SDH) nor FH by IHC although it is recognized that FH IHC is not 100% sensitive. Tumor cells showed widespread membrane positivity for ALK-IHC with positive expression of transcription factor E3 (TFE3)-IHC (Figure 5). Molecular cytogenetic investigations were performed on the 2022 specimen and an ALK rearrangement was confirmed by FISH analysis in 19 of 20 cells examined, with no evidence of TFE3 gene rearrangement by FISH. Further molecular analysis to characterize the exact ALK fusion partner was not undertaken.

Finally, a diagnosis of ALK-RCC was made. Regression of the liver lesions on the restaging scan was noticed three months after the commencement of the ALK inhibitor therapy (alectinib).

## Discussion

The diagnosis of ALK-RCC based on histological features alone is difficult, as its heterogeneous appearance can mimic various renal cell carcinomas. Our case was initially diagnosed following referral to a world-renowned expert as combined tubulocystic and collecting duct renal carcinoma in 2015 based on the immunohistochemical and morphological features and without molecular investigations. Since then, there has been a rapid expansion of molecularly defined renal tumors and a willingness to consider ALK-RCC in the differential of difficult-to-classify renal tumors. ALK-RCC was regarded as an emerging entity in the WHO 2016 classification and became a distinct entity based on worldwide publications in WHO 2022, even though these tumors remain rare.

This report highlights the importance of pathologically reviewing renal tumors that were previously difficult to classify, especially if the clinical behavior is atypical. The patient being alive after seven years was very unusual for collecting duct carcinoma with node-positive disease at initial presentation. It is important to consider novel and emerging entities from the most recent WHO 2022 classification, many of which are defined by molecular characteristics. In our case, this significantly altered the patient’s treatment, leading to the introduction of specific ALK-TKI therapy, with a dramatic effect on the patient’s condition. Clinical studies in non-small cell lung cancer have shown alectinib (an inhibitor of ALK and ALK-variant proteins resulting from rearrangement of the ALK locus) to be an effective therapy in tumors with ALK rearrangements. The identification of ALK rearrangements in other tumor types has subsequently gained importance owing to the availability of ALK inhibitor-targeted therapies [11,12].

To our knowledge, there are only five prior reports of ALK-RCC treated with targeted ALK inhibitor therapy in the literature. A previously published literature review of these cases described outcomes in four patients who received alectinib and one who received crizotinib [13]. All patients treated with alectinib had a radiological partial response and the one patient treated with crizotinib had stable disease. Treatment was ongoing in all patients at the time of publication with durations ranging from three to nine months [13].

## Conclusions

We reported this case to highlight the importance of recognizing and diagnosing this rare RCC subtype since it has significant therapeutic implications. Furthermore, our patient has had a complete radiologic response lasting 12 months so far, which is the longest and most durable response reported to date to our knowledge. A concerted effort by the histopathology and oncology community is needed to gather more data on the incidence and treatment outcomes of these tumors so that sustained progress can be made in optimizing their management.

## References

[1] Shaw AT, Kim DW, Nakagawa K (2013). Crizotinib versus chemotherapy in advanced ALK-positive lung cancer. N Engl J Med.

[2] Brugières L, Cozic N, Houot R (2023). Efficacy and safety of crizotinib in ALK-positive systemic anaplastic large-cell lymphoma in children, adolescents, and adult patients: results of the French AcSé-crizotinib trial. Eur J Cancer.

[3] Shreenivas A, Janku F, Gouda MA, Chen HZ, George B, Kato S, Kurzrock R (2023). ALK fusions in the pan-cancer setting: another tumor-agnostic target?. NPJ Precis Oncol.

[4] Pal SK, Bergerot P, Dizman N (2018). Responses to alectinib in ALK-rearranged papillary renal cell carcinoma. Eur Urol.

[5] Capitanio U, Bensalah K, Bex A (2019). Epidemiology of renal cell carcinoma. Eur Urol.

[6] Moch H, Amin MB, Berney DM (2022). The 2022 World Health Organization Classification of Tumours of the Urinary System and Male Genital Organs-Part A: Renal, Penile, and Testicular Tumours. Eur Urol.

[7] Yang J, Dong L, Du H, Li XB, Liang YX, Liu GR (2019). ALK-TPM3 rearrangement in adult renal cell carcinoma: a case report and literature review. Diagn Pathol.

[8] Doğan K, Onder E (2024). ALK-rearranged renal cell carcinoma (ALK-RCC): evaluation of histomorphological and immunohistochemical features by analysis of 276 renal cell carcinoma cases in Turkey. Pathol Res Pract.

[9] Kuroda N, Trpkov K, Gao Y (2020). ALK rearranged renal cell carcinoma (ALK-RCC): a multi-institutional study of twelve cases with identification of novel partner genes CLIP1, KIF5B and KIAA1217. Mod Pathol.

[10] Debelenko LV, Raimondi SC, Daw N, Shivakumar BR, Huang D, Nelson M, Bridge JA (2011). Renal cell carcinoma with novel VCL-ALK fusion: new representative of ALK-associated tumor spectrum. Mod Pathol.

[11] Sukov WR, Hodge JC, Lohse CM, Akre MK, Leibovich BC, Thompson RH, Cheville JC (2012). ALK alterations in adult renal cell carcinoma: frequency, clinicopathologic features and outcome in a large series of consecutively treated patients. Mod Pathol.

[12] Peters S, Camidge DR, Shaw AT (2017). Alectinib versus crizotinib in untreated ALK-positive non-small-cell lung cancer. N Engl J Med.

[13] Iannantuono GM, Riondino S, Sganga S, Roselli M, Torino F (2022). Activity of ALK inhibitors in renal cancer with ALK alterations: a systematic review. Int J Mol Sci.

